# When the Gallbladder Hurts the Thigh: Silent Gallbladder Empyema With Perforation Presenting as Severe Rhabdomyolysis

**DOI:** 10.7759/cureus.116892

**Published:** 2026-09-25

**Authors:** Enzhong Loo, Kar Hui Khit

**Affiliations:** 1 Acute Internal Medicine, Royal Surrey County Hospital, Guildford, GBR; 2 Internal Medicine and Dermatology, East Surrey Hospital, Redhill, GBR

**Keywords:** acute cholecystitis, eus-guided gallbladder drainage, gallbladder empyema, gallbladder perforation, lumen-apposing metal stent, myositis, rhabdomyolysis

## Abstract

Gallbladder empyema and perforation are severe complications of acute cholecystitis that typically present with abdominal pain, fever, and systemic inflammation. However, clinically silent or atypical presentations may occur, particularly in older patients with diabetes and multiple comorbidities, potentially resulting in delayed diagnosis. This is a case of gallbladder empyema complicated by perforation and biliary leakage that presented predominantly with severe thigh pain and rhabdomyolysis, in the absence of abdominal symptoms.

A 71-year-old man with type 2 diabetes mellitus, peripheral vascular disease, chronic kidney disease, ischaemic heart disease, and heart failure with reduced ejection fraction presented with fever, generalised weakness, severe right thigh pain, and inability to mobilise. He consistently denied abdominal pain. Initial investigations revealed marked systemic inflammation, with a white cell count of 28-32 × 10⁹/L and a C-reactive protein (CRP) of 580-616 mg/L. He had acute kidney injury (peak creatinine 406 μmol/L), hyperphosphataemia, and a markedly raised creatine kinase (CK) of 16,697 U/L, consistent with rhabdomyolysis (McMahon score > 6). MRI of the pelvis and femurs demonstrated bilateral, right-predominant muscle oedema without evidence of an intramuscular collection. Non-contrast CT of the abdomen revealed a markedly distended and inflamed gallbladder with adjacent subcapsular and perihepatic collections suggestive of gallbladder perforation with biliary leakage. Ultrasound-guided drainage of the perihepatic collection yielded bilious fluid. The gallbladder empyema was subsequently managed with endoscopic ultrasound-guided cholecystogastrostomy using a Hot AXIOS lumen-apposing metal stent (Boston Scientific Corp., Natick, MA, USA), allowing drainage of purulent bile. Subsequent endoscopic washout was performed, with placement of two pigtail stents through the AXIOS stent. Following antibiotic treatment and biliary source control, inflammatory markers, CK, and renal function improved, with resolution of the thigh pain and improvement in mobility, allowing discharge home.

Gallbladder empyema complicated by perforation can occur without abdominal pain and may present predominantly with extra-abdominal features. In older patients with diabetes and multiple comorbidities who present with unexplained sepsis, an occult intra-abdominal source should be considered even when abdominal symptoms and signs are absent. Although the direct mechanism linking biliary infection to rhabdomyolysis remains uncertain, the parallel resolution of sepsis, CK, renal function, and thigh pain after antibiotic treatment and biliary drainage supports the infection as the unifying driver. This case also demonstrates that endoscopic ultrasound-guided gallbladder drainage with a lumen-apposing metal stent offers an effective and minimally invasive alternative to surgery for source control in patients with high operative risk.

## Introduction

Acute cholecystitis is a common condition; however, progression to gallbladder empyema, gangrene, or perforation is associated with significant morbidity. The Tokyo Guidelines of acute cholecystitis emphasise the integration of clinical findings, laboratory evidence of systemic inflammation, and imaging to establish the diagnosis and assess disease severity, with severe disease defined by the presence of associated organ dysfunction [1]. Although right upper quadrant abdominal pain, fever, and a positive Murphy’s sign are characteristic features of acute cholecystitis [1], the severity of the underlying gallbladder pathology may not always be accurately reflected by the clinical presentation [2]. Importantly, several case reports have described complicated gallbladder infections, including empyema, gangrenous cholecystitis, and perforation presenting with minimal or absent abdominal symptoms, potentially leading to delayed or missed diagnoses [3-8].

Rhabdomyolysis is characterised by acute skeletal muscle injury with release of intracellular contents, particularly creatine kinase (CK), and may result in acute kidney injury. Established causes include trauma, prolonged immobilisation, muscle ischaemia, medications, metabolic disorders, and systemic infections [9,10]. The McMahon Score for rhabdomyolysis is a validated risk-prediction tool that incorporates clinical and laboratory parameters at presentation, including age, sex, initial creatinine, calcium, phosphate, bicarbonate, CK level, and aetiology to estimate the risk of renal replacement therapy (RRT) or in-hospital mortality in patients with rhabdomyolysis [11]. A score of >10 identifies patients at high risk of RRT or in-hospital mortality and may facilitate early risk stratification and management [11].

This case highlights the diagnostic challenge of severe intra-abdominal infection presenting predominantly with extra-abdominal manifestations, in which perforated gallbladder empyema occurred in the absence of abdominal symptoms. When accompanied by severe muscle pain and marked CK elevation, rhabdomyolysis may initially suggest a primary musculoskeletal process, potentially diverting attention from an underlying systemic or extra-muscular source of infection. Although no previously published case report has specifically described rhabdomyolysis secondary to gallbladder empyema or perforation, rhabdomyolysis has been reported as an uncommon complication of severe intra-abdominal infections [12-14]. These reports suggest that severe intra-abdominal infection, including complicated biliary infection, may precipitate rhabdomyolysis in the setting of systemic inflammation and multiorgan dysfunction.

## Case presentation

A 71-year-old obese man presented with a five-day history of general malaise, fever, progressive weakness, and severe right thigh pain with significant difficulty mobilising. He denied abdominal pain and reported no chest pain, back pain, loin pain, urinary symptoms, vomiting, or diarrhoea. There was no preceding trauma or unusual physical exertion. Two months earlier, he had undergone left distal first metatarsal amputation for diabetic foot infection and osteomyelitis. The amputation site was clinically well healed with no evidence of ongoing infection.

His medical history included type 2 diabetes mellitus complicated by peripheral neuropathy and retinopathy, hypertension, peripheral vascular disease with a previous left popliteal artery occlusion treated with angioplasty, ischaemic heart disease with severe multivessel coronary artery disease, heart failure with reduced ejection fraction (30%-35%), and chronic kidney disease stage G3b, with a baseline creatinine of approximately 180-200 μmol/L and an estimated glomerular filtration rate (eGFR) of 30-35 mL/min/1.73 m². His regular medications were insulin, linagliptin, aspirin, clopidogrel, lisinopril, furosemide, amitriptyline, pravastatin 20 mg nightly, and ezetimibe 10 mg daily. Pravastatin was started three months ago.

On admission (day 1), he was febrile at 38.4°C. Blood pressure was 130/75 mmHg, heart rate was 97 bpm, and oxygen saturation was 96% on room air, with no episodes of hypotension during admission. Physical examination was unremarkable apart from marked tenderness over the right thigh, with hip movement limited by pain. There were no overlying skin changes such as erythema or necrosis, and no palpable collection, or clinical evidence of compartment syndrome. The left foot amputation site appeared healthy, without cellulitis or abnormal discharge. Abdominal examination revealed a soft, non-tender abdomen with a negative Murphy’s sign. X-rays of the right hip/femur and both feet were unremarkable.

Initial blood investigations demonstrated a profound systemic inflammatory response, marked CK elevation, acute-on-chronic kidney injury, and hyperphosphataemia (Table 1). The McMahon score for rhabdomyolysis was 10. Extended autoimmune and myositis antibody panels were negative (Table 2), and multiple blood cultures showed no growth.

**Table 1:** Serial blood investigations eGFR: estimated glomerular filtration rate

	Day 1	Day 2	Day 2	Day 3	Day 3	Day 4	Day 4	day 4	Day 5	Day 6	Day 7	Day 8	Day 9	Day 10	Day 11	Day 12	Day 13	Day 14	Reference range
White cell count (×10⁹/L)	28.2	32.3	29.7	23.7	22	22.5	19.2	20.2	17.7		18.7	16.4	18.9	21.4	16	16.9	14.9	14.8	4-11
Haemoglobin (g/L)	123	128	129	113	112	115	110	116	109		106	106	112	93	90	86	93	93	130-180
Platelets (×10⁹/L)	284	292	307	282	300	323	307	349	321		395	424	494	526	528	544	627	623	150-450
Neutrophils (×10⁹/L)	25.1	29.2	27.1	21.3	19.7	19.8	16.7	17.9	15.2		15.5	13.1	16.2	18.3	13.1	13.7	12.2	12.2	2.0-7.5
Sodium (mmol/L)	134	134	132	136	134	137	137	137	137	138	136	140	141	139	140	141	139	141	133-146
Potassium (mmol/L)	4.7	4.8	5.1	4.4	4.2	4.1	4.2	4.2	4.4	4.3	4.5	4.7	5.4	5.1	4.9	4.6	5.4	5.5	3.5-5.3
Urea (mmol/L)	14.1	20	22.4	26.7	27.3	29.5	29.2	28.5	26.1	20.8	17.1	12.5	10.7	10.6	9.8	7.1	5.1	5.2	2.5-7.8
Creatinine (µmol/L)	252	323	361	406	398	392	368	349	297	252	211	197	194	205	194	159	157	168	64-104
eGFR (mL/min/1.73 m²)	21	16	14	12	12	12	13	14	17	21	26	29	29	27	29	37	38	35	90-120
Albumin (g/L)	32		27	22	22	22	21	21	19	18	18	19	21	18	18	18	20	21	35-50
Total bilirubin (µmol/L)	18		17	14		13	12	14	13	8	9	8	10	9	7	5	5	5	0-20
Alkaline phosphatase (U/L)	133		184	169	331	203	204	235	262	254	336	408	544	481	528	464	447	399	49-140
Alanine transaminase (U/L)	42		46	34		46	47	57	49	42	35	44	64	249	163	124	85	53	0-55
Aspartate transaminase (U/L)										52									0-34
Creatine kinase (U/L)	16,697	9,506	6,136	2,743	2,476		1,453	1,044	468	296	184	104	110						40-320
Calcium (mmol/L)	2.45		2.42	2.23	2.26	2.24	2.17		2.29	2.16	2.15	2.23	2.18	2.34	2.38	2.18	2.34		2.2-2.6
Phosphate (mmol/L)	1.35		2.26	2.52	2.25	2.22	2.03		1.54	1.28	0.99	0.86	0.9	1.02	1	0.91	1.05		0.8-1.5
Magnesium (mmol/L)			1.06	1.12		1.16	1.14		1.1	1.07	0.87	0.86	0.81	0.84	0.84	0.84			0.7-1.0
C-reactive protein (mg/L)	442	580	616	480	505	500	442	436	401	309	204	168	171	334	280	145	90	94	0-5

**Table 2:** Extended autoimmune and myositis antibody panels

Autoimmune and myositis antibody panels	Results	Reference range
Antinuclear antibodies (ANA)	<0.1	<0.1
Extractable nuclear antigen antibodies (ENA)	<0.1	<0.1
Anti-myeloperoxidase antibodies (MPO Ab)	<0.2	<0.2
Anti-proteinase 3 antibodies (PR3 Ab)	<0.6	<0.6
Anti-glomerular basement membrane antibodies (GBM Ab)	<2	<2
Anti-Mi-2 alpha antibodies (Mi-2α Ab)	Negative	Negative
Anti-Mi-2 beta antibodies (Mi-2β Ab)	Negative	Negative
Anti-transcriptional intermediary factor 1-gamma antibodies (TIF1-γ Ab)	Negative	Negative
Anti-melanoma differentiation-associated protein 5 antibodies (MDA5 Ab)	Negative	Negative
Anti-nuclear matrix protein 2 antibodies (NXP-2 Ab)	Negative	Negative
Anti-small ubiquitin-like modifier activating enzyme 1 antibodies (SAE-1 Ab)	Negative	Negative
Anti-Ku antibodies (Ku Ab)	Negative	Negative
Anti-polymyositis/scleroderma 100 antibodies (PM-Scl 100 Ab)	Negative	Negative
Anti-polymyositis/scleroderma 75 antibodies (PM-Scl 75 Ab)	Negative	Negative
Anti-histidyl-tRNA synthetase antibodies (Jo-1 Ab)	Negative	Negative
Anti-signal recognition particle antibodies (SRP Ab)	Negative	Negative
Anti-threonyl-tRNA synthetase antibodies (PL-7 Ab)	Negative	Negative
Anti-alanyl-tRNA synthetase antibodies (PL-12 Ab)	Negative	Negative
Anti-glycyl-tRNA synthetase antibodies (EJ Ab)	Negative	Negative
Anti-isoleucyl-tRNA synthetase antibodies (OJ Ab)	Negative	Negative
Anti-Ro52 (TRIM21) antibodies (Ro-52 Ab)	Negative	Negative
Anti-3-hydroxy-3-methylglutaryl-coenzyme A reductase antibodies (HMGCR Ab)	<1.5	<1.5

MRI of the pelvis and femurs (Figure 1) demonstrated extensive muscle oedema involving the right gluteus medius, piriformis, adductor magnus, biceps femoris, and semimembranosus muscles, with smaller areas of involvement on the left. There was no intramuscular collection, perifascial fluid, or subcutaneous oedema. These appearances were considered more consistent with a reactive or systemic process than with primary pyomyositis. In view of the marked CK elevation, pravastatin and ezetimibe were withheld.

**Figure 1:**
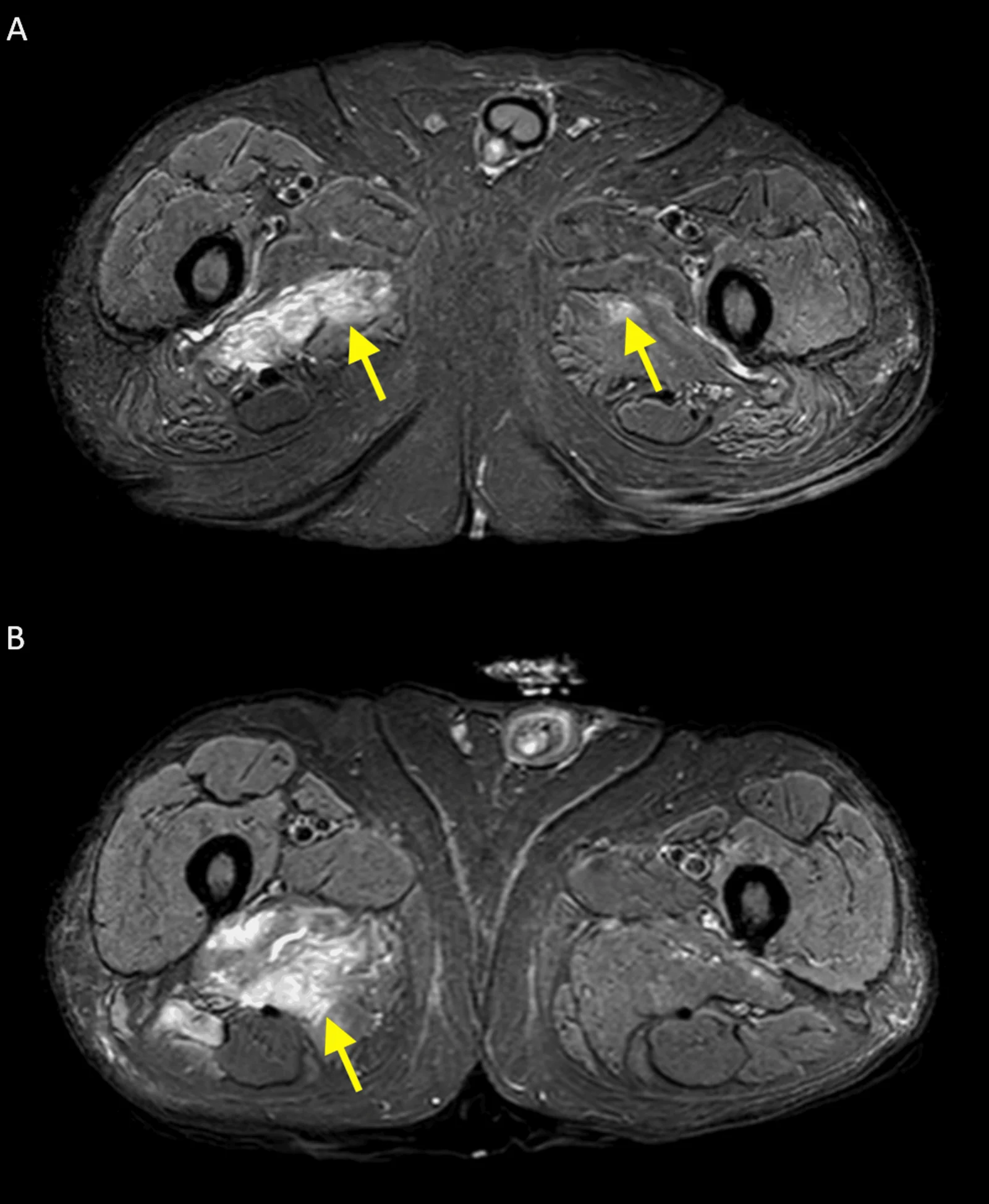
(A, B) MRI pelvis and femurs STIR TSE axial. There are large areas of muscle oedema (yellow arrows) involving the right gluteus medius, piriformis, adductor magnus, biceps femoris, and semimembranosus. On the left, there are small foci of muscle oedema within the obturator internus, adductor magnus, and biceps femoris. There is no intramuscular collection, perifascial fluid, or subcutaneous oedema. STIR: short tau inversion recovery; TSE: turbo spin echo

Given the severity of the systemic inflammatory response and the absence of a clear infective source, further investigation was undertaken. Non-contrast CT of the abdomen (Figure 2) on day 3 of admission demonstrated a markedly distended gallbladder containing high-density layering material, with localised pericholecystic inflammatory changes and a loculated subcapsular and perihepatic fluid collection extending towards the right paracolic gutter. The overall radiological findings were suggestive of acute cholecystitis complicated by possible gallbladder rupture and an associated biliary collection.

**Figure 2:**
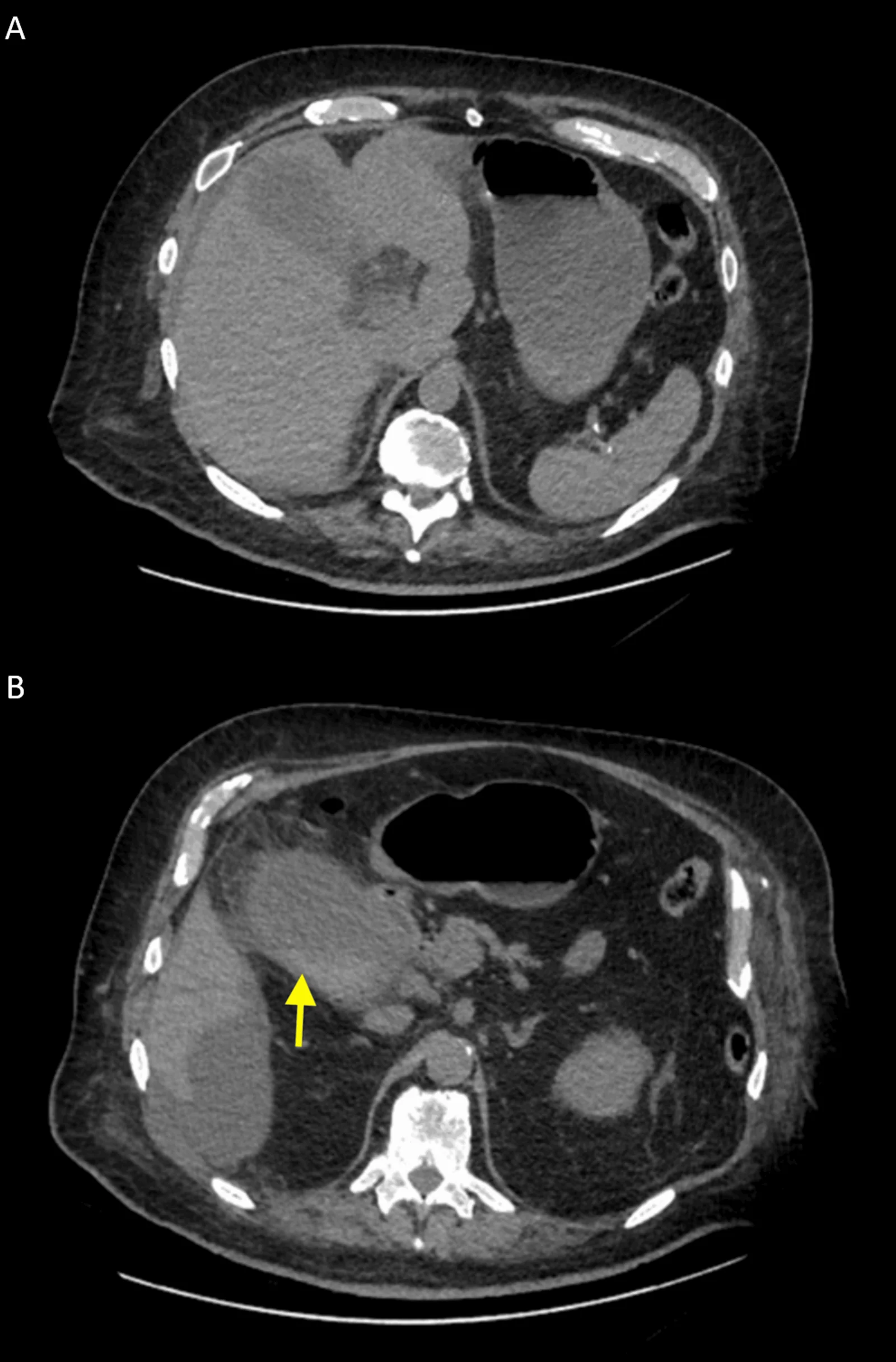
(A, B) CT abdomen and pelvis non-contrast. The gallbladder is distended (yellow arrow) with localised inflammatory changes suggestive of cholecystitis. Perihepatic fluid may represent a biliary collection secondary to gallbladder rupture. Loculated subcapsular hepatic fluid is seen, extending into the right paracolic gutter. No evidence of any further abdominal or pelvic collections.

The patient was initially treated with intravenous fluids and empirical flucloxacillin and clindamycin for suspected pyomyositis. This was changed to piperacillin-tazobactam once biliary sepsis became the leading diagnosis. On day 4 of admission, an abdominal ultrasound was performed. Although technically limited, it confirmed gallbladder distension and the presence of fluid inferior to the right hepatic lobe. An 8 Fr locking pigtail drain was inserted into the perihepatic collection under ultrasound guidance, yielding approximately 1 L of bilious fluid. The drain subsequently required wire exchange and upsizing due to obstruction. Percutaneous cholecystostomy was avoided because of the high bleeding risk associated with dual antiplatelet therapy (aspirin and clopidogrel) and the need to traverse hepatic parenchyma. As the perihepatic collection was thought to communicate with the gallbladder, a safer ultrasound-guided drainage of this collection was performed instead. Perihepatic fluid analysis showed inflammatory cells, predominantly polymorphs, with no organisms or microbial growth.

Despite improvement in inflammatory markers, the gallbladder remained markedly distended, measuring 10.7 × 10 cm on repeat contrast-enhanced CT performed on day 9 of admission (Figure 3). Given his prohibitive operative risk, a minimally invasive approach to definitive gallbladder drainage was favoured. This was deferred until day 9 of admission because dual antiplatelet therapy had been restarted after the initial ultrasound-guided drainage, and clopidogrel would have needed to be stopped for at least five days. Endoscopic ultrasound (EUS)-guided cholecystogastrostomy was performed using a Hot AXIOS lumen-apposing metal stent (Boston Scientific Corp., Natick, MA, USA) (Figure 4), deployed through an avascular tract of the gastric antrum. More than 200 mL of purulent bile was drained. Residual intraluminal debris and concern regarding stent patency prompted a further endoscopic washout and placement of two pigtail stents through the AXIOS stent. Bile cultures subsequently grew *Escherichia coli* and *Enterococcus faecium*.

**Figure 3:**
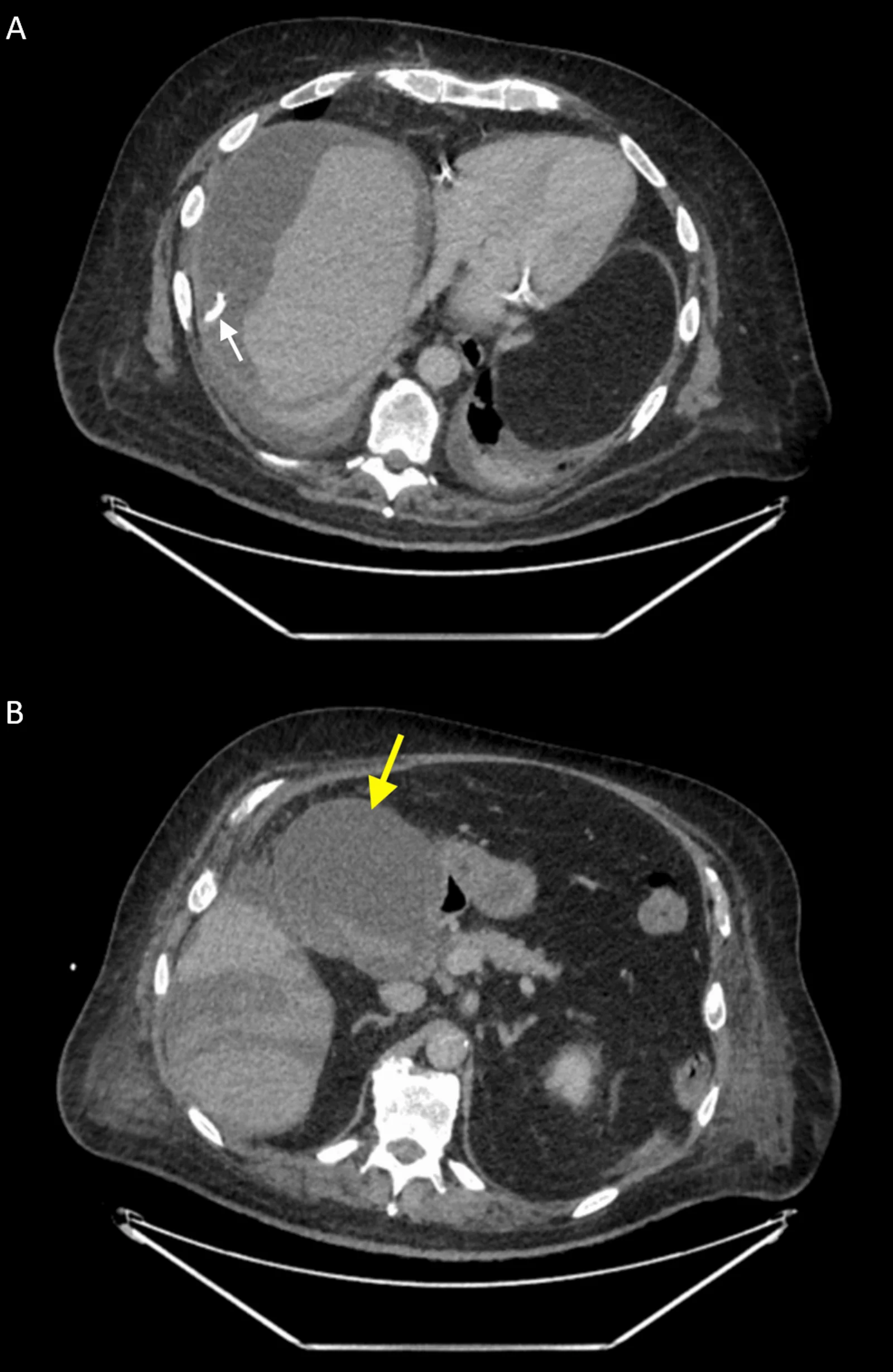
(A, B) CT abdomen and pelvis with contrast. The gallbladder is distended (yellow arrow) with mild pericholecystic fat stranding. Some hyperattenuating material is seen near the neck of the gallbladder, likely representing gallstones. A perihepatic pigtail drain is in situ (white arrow), with its distal end positioned adjacent to segments 6 and 7.

**Figure 4:**
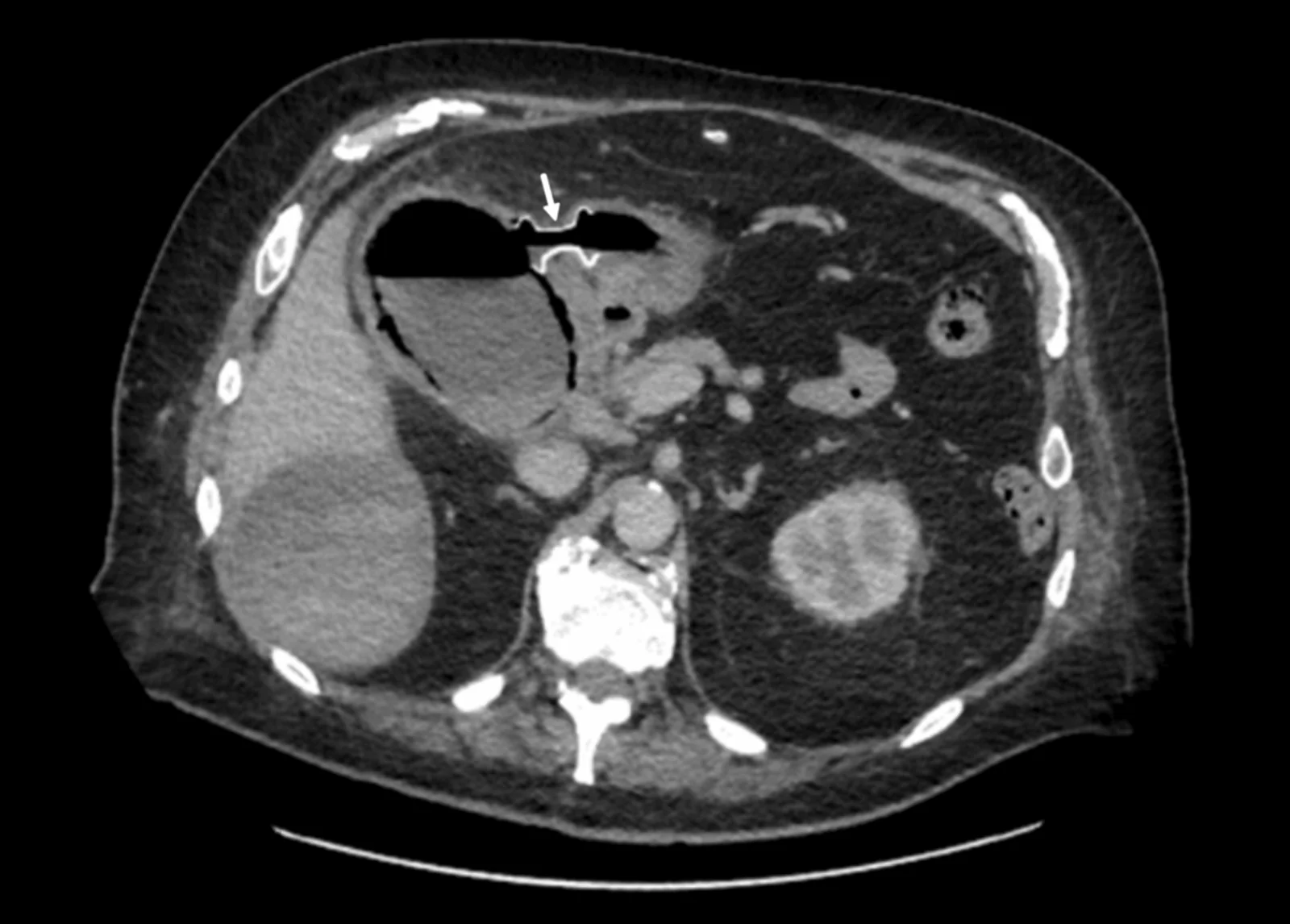
CT abdomen and pelvis with contrast. The distended gallbladder following placement of a Hot AXIOS lumen-apposing metal stent (white arrow), creating a cholecystogastrostomy.

Following definitive source control and antibiotic therapy, CK fell rapidly and subsequently normalised, with concomitant improvement in renal function and inflammatory markers. His thigh pain resolved, with progressive improvement in mobility. Notably, he remained free of abdominal pain throughout the admission and was discharged home with outpatient follow-up.

## Discussion

This case demonstrates that complicated gallbladder infection can present with a predominantly extra-abdominal phenotype. To our knowledge, rhabdomyolysis secondary to gallbladder empyema or perforation has not previously been described in the literature. The absence of severe abdominal pain is recognised in some cases of complicated cholecystitis [3-8]. The combination of severe thigh pain, a CK level of 16,697 U/L, extensive muscle oedema, and marked systemic inflammation initially raised the possibility of pyomyositis, potentially from haematogenous seeding of the recent diabetic foot infection, as well as statin-associated myopathy, idiopathic inflammatory myopathy, or multifactorial rhabdomyolysis. However, the absence of a focal intramuscular collection on MRI, negative myositis serology, lack of preceding muscle symptoms or rash, and parallel improvement in the muscle abnormalities following biliary source control made a primary inflammatory or infectious myopathy unlikely.

Systemic infection is a recognised precipitant of rhabdomyolysis [9]. In this patient, the aetiology of rhabdomyolysis was likely multifactorial, with gallbladder empyema acting as a potential trigger. Several plausible pathophysiological mechanisms may have converged to precipitate muscle injury. His background of peripheral vascular disease with chronic arterial insufficiency, compounded by impaired tissue perfusion during sepsis, may have exacerbated skeletal muscle ischaemia. Furthermore, his concurrent chronic kidney disease may have impaired the clearance of circulating myotoxins and inflammatory mediators, thereby compounding the systemic metabolic insult. Other potential contributors to the elevated CK level included prolonged immobility and statin therapy. Statin-associated myopathy was considered in the differential diagnosis. The patient had been started on low-intensity pravastatin 20 mg three months before admission and had experienced no preceding muscle symptoms. The presence of high fever, a marked inflammatory response, negative anti-HMGCR antibodies, and clear temporal improvement following biliary source control made isolated statin-associated myopathy unlikely. This multifactorial interplay, together with the clinical trajectory characterised by rapid improvement following antibiotic therapy and biliary drainage, further supports occult biliary sepsis as the principal driver of the presentation.

Ultrasonography remains an important first-line investigation in complicated cholecystitis and may identify features suggestive of gallbladder empyema, including gallbladder distension, wall thickening, pericholecystic fluid, and intraluminal echogenic material [15]. CT is better than ultrasound for demonstrating gallbladder perforation, more frequently identifying the site of wall disruption or focal bulging (69% vs 38% with ultrasound), whilst also better delineating associated pericholecystic fluid collections and abscesses [16].

Source control presented a further challenge in this patient given the high operative risk. Percutaneous ultrasound-guided drainage of the perihepatic collection provided initial clinical improvement but did not achieve definitive source control with persistent gallbladder empyema. EUS-guided gallbladder drainage using a lumen-apposing metal stent has emerged as an effective minimally invasive alternative for patients who are unsuitable for cholecystectomy, with high technical and clinical success rates [17-20]. In the DRAC-1 randomised trial, EUS-guided gallbladder drainage was associated with fewer adverse events and reinterventions than percutaneous cholecystostomy in very high-risk surgical patients with acute cholecystitis [18]. In this case, cholecystogastrostomy followed by endoscopic washout and placement of pigtail stents through the lumen-apposing metal stent achieved effective and sustained internal drainage.

## Conclusions

This case highlights the importance of reconsidering an apparent musculoskeletal diagnosis when the degree of systemic inflammation is disproportionate to the clinical findings. Muscle oedema and rhabdomyolysis may represent secondary manifestations of an occult systemic infection, even in the absence of localising symptoms. In patients with severe sepsis and an unexplained musculoskeletal presentation, cross-sectional imaging may therefore be crucial in identifying a treatable intra-abdominal source of sepsis. Where surgery carries prohibitive risk, EUS-guided gallbladder drainage provides a minimally invasive approach for definitive source control.
